# Predominant VH1-69 IgBCR Clones Show Higher Expression of CD5 in Heterogeneous Chronic Lymphocytic Leukemia Populations

**DOI:** 10.3389/fonc.2021.703254

**Published:** 2021-06-18

**Authors:** Domenico Maisano, Enrico Iaccino, Alessandro D’Ambrosio, Federico Chiurazzi, Vincenzo Dattilo, Mariangela Scalise, Massimo Gentile, Eleonora Vecchio, Nancy Nisticò, Annamaria Aloisio, Erika De Sensi, Giuseppe Fiume, Ileana Quinto, Selena Mimmi

**Affiliations:** ^1^ Laboratory of Immunology, Department of Experimental and Clinical Medicine, University Magna Graecia of Catanzaro, Catanzaro, Italy; ^2^ Hematological Clinic, Department of Clinical Medicine, University “Federico II” of Naples, Naples, Italy; ^3^ Genetics Unit, IRCCS Centro San Giovanni di Dio Fatebenefratelli, Brescia, Italy; ^4^ Laboratory of Molecular and Cellular Cardiology, Department of Experimental and Clinical Medicine, University Magna Graecia of Catanzaro, Catanzaro, Italy; ^5^ Hematology Unit, AO, Cosenza, Italy

**Keywords:** chronic lymphocytic leukemia, phage display, immunoglobulin B cell receptor, peptide-based sorting, gene expression

## Abstract

The immunoglobulin B cell receptor (IgBCR) expressed by chronic lymphocytic leukemia (CLL) B cells plays a pivotal role in tumorigenesis, supporting neoplastic transformation, survival, and expansion of tumor clones. We demonstrated that in the same patient, two or more CLL clones could coexist, recognized by the expression of different variable regions of the heavy chain of IgBCR, composing the antigen-binding site. In this regard, phage display screening could be considered the easier and most advantageous methodology for the identification of small peptide molecules able to mimic the natural antigen of the tumor IgBCRs. These molecules, properly functionalized, could be used as a probe to specifically identify and isolate single CLL subpopulations, for a deeper analysis in terms of drug resistance, phenotype, and gene expression. Furthermore, CLL cells express another surface membrane receptor, the CD5, which is commonly expressed by normal T cells. Piece of evidence supports a possible contribution of CD5 to the selection and maintenance of autoreactivity in B cells and the constitutive expression of CD5 on CLL cells could induce pro-survival stimuli. In this brief research report, we describe a peptide-based single-cell sorting using as bait the IgBCR of tumor cells; in the next step, we performed a quantitative analysis of CD5 expression by qRT-PCR related to the expressed IgBCR. Our approach could open a new perspective for the identification, isolation, and investigation of all subsets of IgBCR-related CLL clones, with particular attention to the more aggressive clones.

## Introduction

CD5 is a membrane surface receptor expressed by thymocytes, mature T cells, B1a subset of B cells, and leukemic B cells of chronic lymphocytic leukemia (B-CLL) disease (1, 2). Called also Leu-1, it is a 67-kDa type I transmembrane glycoprotein monomer and a member of the scavenger receptor cysteine-rich (SRCR) family (3). The extracellular region is composed of three different domains (D1, D2, and D3) and represents the putative binding region, while the intracellular domain contains the Immunoreceptor Tyrosine-based Activation Motif (ITAM) sequence as the docking site for phosphorylated Src homology 2 (SH2) domain-containing proteins (4).

CD5 is not expressed in normal B cells, except the B1 subgroup, while it is mostly expressed in B-CLL cells (2); this suggests a possible critical role of CD5 in self-maintenance and progression of neoplastic B cells (5, 6). This hypothesis is supported by the evidence that CD5 activates multiple signaling pathways, including mitogen-activated protein kinase (MAPK) (Ras/Erk) pathway, the Ca2+–calmodulin–calcineurin–NFAT pathway, and the PI3-K/Akt/mTOR pathway (7). Further, in transgenic mice, the expression of CD5 correlates with the self-reactivity in B cell populations, supporting a possible contribution to the selection and maintenance of autoreactivity in B cells (8).

CLL is the most frequent adult leukemia in western countries, with a variable clinical course and the occurrence of a heterogeneous tumor population (9, 10). Increasing evidence supports the hypothesis that CLL pathogenesis is an antigen-driven process by a continuous triggering of the immunoglobulin B cell receptor (IgBCR) (11–15), resulting in the no random choice of heavy chain variable region (VH) family during B cell development and the consequential expression of the stereotyped IgBCRs, frequently found in different patients (16). The expression of peculiar IgBCRs is often related to the aggressiveness of the disease (17). Indeed, the unmutated CLL (U-CLL), with less than 2% of mutation in comparison with the germline sequence, seems to be more aggressive with respect to the mutated CLL (M-CLL), which shows a higher percentage of mutations in the variable region of the heavy chain (18). Furthermore, in the U-CLL subgroup, the rearrangement VH1-69 is the most representative (about 25%), and patients showed an aggressive disease with the expansion of CLL clones expressing the unmutated IgBCRs, drug resistance, and often a fatal outcome (16, 19).

Since CD5 seems to be located close to the surface IgBCR on the B cell surface (6) and seems to be a potential ligand of peculiar Ig heavy chain framework sequences in malignant B cells (5), these findings suggest that CD5 could be a self-antigen recognized by the CLL-IgBCRs, promoting survival and proliferation signaling.

In our last published work, we analyzed two CLL patients (named CLL1 and CLL5) for 2-years observation, demonstrating the coexisting of several leukemic subpopulations identified by different IgBCRs, but the most representative subpopulation identified by the rearrangement VH1-69 persisted during all the time (20). So, based on the evidence mentioned above, we asked whether the survival and progression of the VH1-69 subpopulation could be related to higher CD5 gene expression levels compared to the other coexisting clones. Taking advantage of the previously selected peptide (named p1), able to specifically target the leukemic cells expressing the rearrangement VH1-69 (20), we performed a peptide-based cell sorting, in order to isolate the VH1-69 clones from peripheral blood of both oligoclonal CLL1 and CLL5 patients. p1 positive sorted clones (corresponding to the VH1-69 clones) were analyzed by qRT-PCR for the expression of CD5, compared to the other CLL clones (p1 negative clones).

Our results demonstrate that this approach, extended to all IgBCR subsets, could open new strategies for a deeper comprehension of the most aggressive clones, analyzing a wide range of molecular mechanisms and drug resistance - related genes.

## Methods

### Peptide-Based Cell Sorting

Frozen B-CLL cells previously isolated from CLL patients were gently thawed and 24h-cultured in RPMI medium supplemented with 10% fetal bovine serum. Then, cells (1 × 10^7^ cells) were first labelled with anti CD19-APC (Miltenyi Biotec, Germany, cat.n. 170-078-090) and anti-CD5-PE. (Miltenyi Biotec – Germany, cat.n. 130-110-990) antibodies, for setting the gate of B-CLL cells. Bulk CLL cells were incubated with FITC-conjugated peptide p1 (1 ng/ml) at 4°C for 20 min, analyzed by flow cytometry, and p1-positive cells were sorted by BD FACSAria III TM (Becton Dickinson). Gating was done using the BD FACSDiva™ software (Becton, Dickinson Biosciences). Cell sorting was performed with 70-μm nozzle size and sorted directly into 5-ml tubes containing 3 ml of staining media in order to minimize cellular stress. Cells were gated in FSC-A vs SSC-A and single cells gated in FSC-H vs FSC-A. Stringent gating strategies to exclude debris or dead cells that exhibit autofluorescence and CD5 negative cells were applied.

### qRT-PCR for CD5 Gene Expression

Total mRNA was extracted from CLL cells (bulk or sorted cells) by TRIzol RNA Isolation Reagents (Invitrogen) according to the manufacturer’s instructions and quantified by spectrophotometer. 500ng of total mRNA was retrotranscribed into cDNA using the iScript™ cDNA Synthesis Kit (BioRad).

qRT-PCR was performed using the CD5 primers (forward 5′ CAGAAGAAGCAGCGCCAGT 3′; reverse 5′ TCCTGGGAGGTTGGCTGTATT 3′). The general reaction conditions were as follows: initial denaturation step at 95°C for 10 min; 40 cycles of denaturation at 95°C for 10 s; annealing at 57°C for 10 s; and elongation at 72°C. All reactions were performed in triplicate employing the CFX96 Touch Deep Well Real-Time PCR System (BioRad). The results normalized to the GAPDH housekeeping gene and determined by ΔΔCt method were represented as log10 fold expression ± SD of triplicate assessments. Statistical significance was evaluated using one-way analysis of variance (ANOVA), followed by Bonferroni’s test for multiple comparisons. Bars show mean values ± 95% confidence intervals based on three biological replications.

## Results

**Figure 1:**
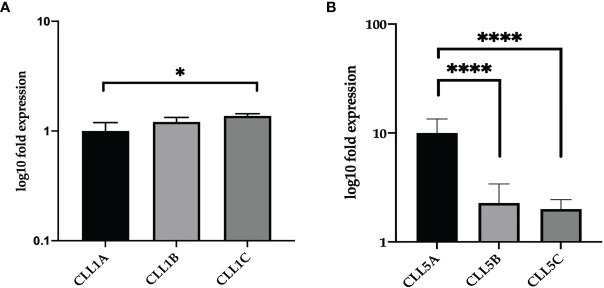
Relative expression of CD5 in CLL clones of CLL1 and CLL5 patients. Relative CD5 gene expression in total B-CLL cells from CLL1 **(A)** and CLL5 **(B)** patient. The results were normalized to the GAPDH housekeeping gene, determined by ΔΔCt method, and represented as log10 fold expression ± SD of triplicate assessments. Statistical significance was evaluated using one-way analysis of variance (ANOVA), followed by Bonferroni’s test for multiple comparisons. Bars show mean values ± 95% confidence intervals based on three biological replications. *P ≤ 0.01; ****P ≤ 0.0001.

The two CLL patients (named CLL1 and CLL5) were previously analyzed on 2 years observation (20). In particular, the patient CLL1 was analyzed at Binet stage A at months 1 and 5 (CLL1A and CLL1B, respectively) and Binet stage C at month 8 (CLL1C); the patient CLL5, being Binet stage A all the time, was analyzed at months 1, 12, and 24 (CLL5A, CLL5B, and CLL5C, respectively) (**Table 1**). As reported by **Table 1**, the VH1-69 subpopulation persisted all the time, at different percentages of representativeness with respect to the other leukemic clones, related to the aggressiveness of the disease. We firstly analyzed by qRT-PCR the CD5 expression levels in total CD5 positive B-CLL cells of CLL1 and CLL5 patients at different times of disease. As shown in **Figure 1A**, relative gene expression of CD5 was slightly increased, but not statistically significant comparing CLL1A to CLL1B (both Binet stage A). However, a significant increase in CD5 expression was associated with the expansion of VH1-69 clone in the passage from Binet stage A to C (CLL1A fold 1.00±0.19; CLL1C fold 1.37±0.06), passing from 50% to 60% up to 80% of representativeness. Differently, in CLL5 patient CD5 expression levels significantly decreased comparing CLL5A and CLL5B, associated to a representativeness decrease of VH1-69 clone, passing from 75% to 35%; further, relative gene expression of CD5 was slightly decreased, but not statistically significant comparing CLL5B to CLL5C (**Figure 1B**). These observations suggested that the expression level of CD5 correlated with the percentage of the existing VH1-69 CLL clones.

**Table 1:** Clinical and molecular data of CLL1 and CLL5 patients.

Patient	Sample (collection time)	WBC (% of CD19/CD5 positive)	Binet stage	VH1-69 subpopulation/total CLL cells (%)
CLL1 65-years old male	CLL1A (month 1)	40,410/mmc (90%)	A	60%
	CLL1B (month 5)	69,070/mmc (92%)	A	50%
	CLL1C (month 8)	92,670/mmc (99%)	C	80%
CLL5 80-years old female	CLL5A (month 1)	57,210/mmc (95%)	A	75%
	CLL5B (month 12)	119,999/mmc (98%)	A	45%
	CLL5C (month 24)	86,500/mmc (96%)	A	35%

Peripheral blood samples of CLL1 and CLL5 patients were collected at the indicated time. CLL stage was defined according to Binet classification (Cancer.Net Editorial Board, 10/2017). The percentage of VH1-69 subpopulation cells was determined by IgBCR sequencing as previously reported (20). The nucleotide sequences of CLL IgBCRs were deposited (GenBank accession numbers MT334403 to MT334414).

**Figure 2:**
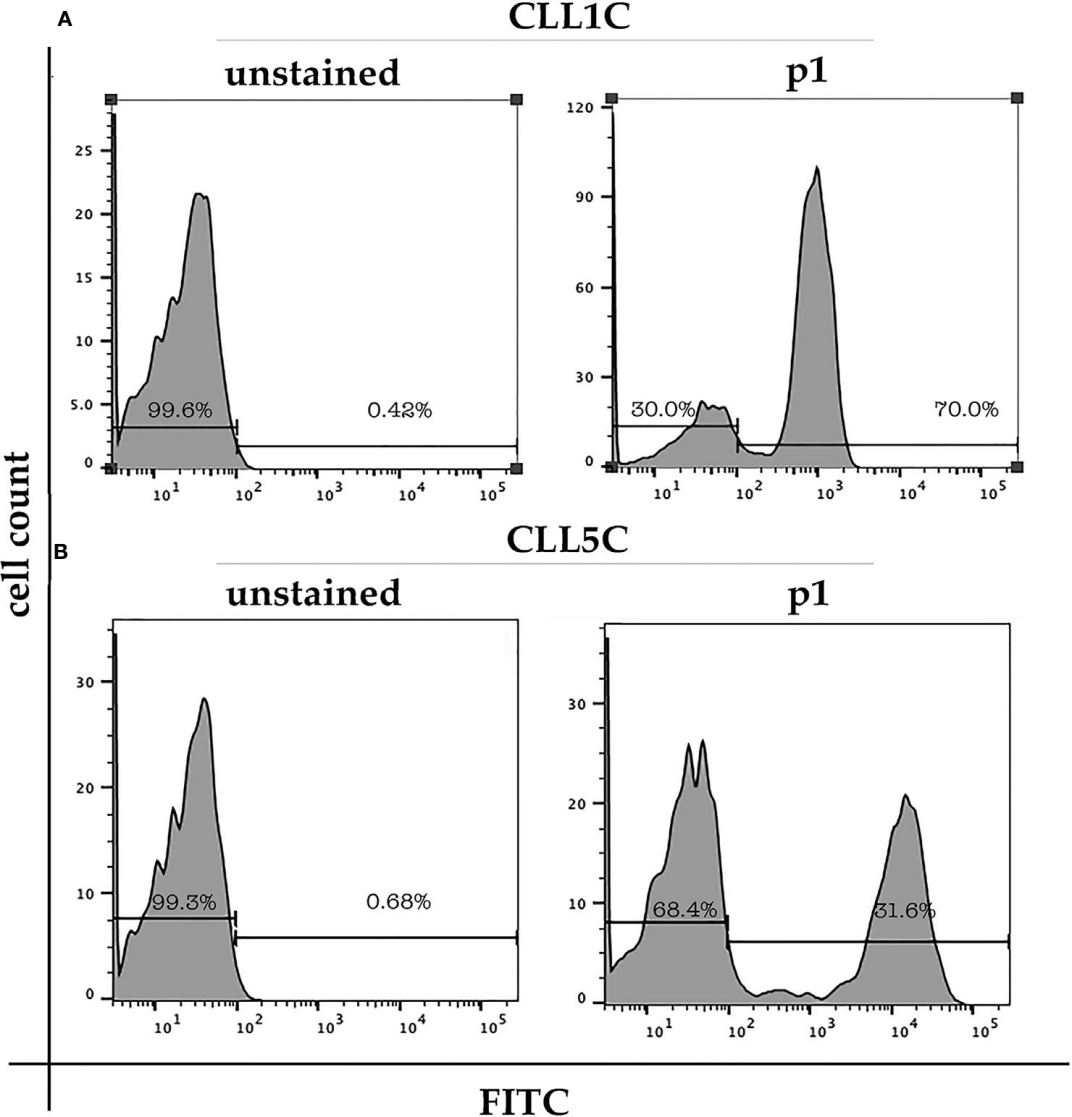
CD19/CD5 positive B-CLL cells isolated from CLL1C (month 8, panel **A**) and CLL5C (month 24, panel **B**) patients were stained with FITC-conjugated peptide p1 or unstained. p1-positive cells corresponding to the VH 1-69 clones were 70% of total B-CLL population in CLL1C patient and 32% in CLL5C patient.

To deepen our analysis, we took advantage of the previously identified peptide p1 as a specific ligand of the VH1-69 unmutated subpopulation in CLL1 and CLL5 patients (20). Indeed, in this work, we used the peptide p1 as a probe to sort the VH1-69 subpopulation from the total B-CLL cells of CLL1 and CLL5 patients. **Figure 2** shows the coexisting of a p1 positive and a p1 negative population in the total CLL cells of CLL1 (**Figure 2A**) and CLL5 (**Figure 2B**) patients. After peptide-based sorting, IgBCRs sequence was analyzed both in p1-positive and p1-negative fraction, to validate the sorting procedures. No VH1-69 rearrangement was found in the p1-negative fraction.

**Figure 3:**
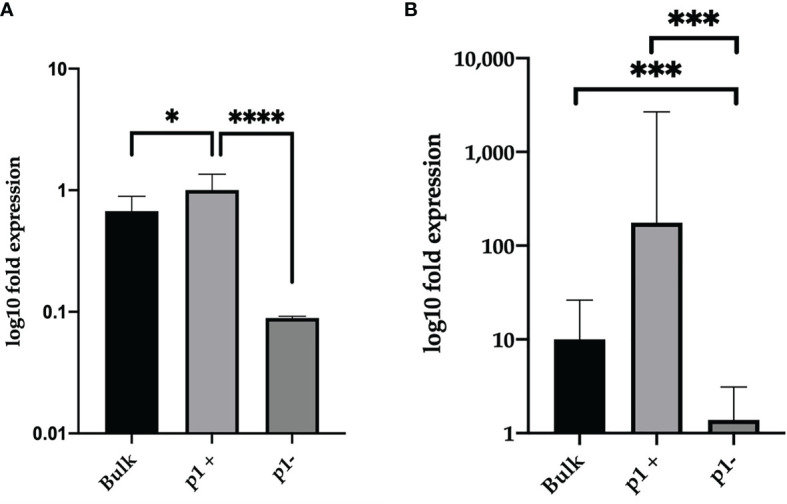
Relative expression of CD5 in p1 positive and p1 negative CLL clones of CLL1 and CLL5 patients. Relative CD5 gene expression in B-CLL subpopulations of CLL1C **(A)** and CLL5C **(B)** sample. Bulk represents the total CLL population, p1-positive the sorted VH1-69 CLL cells, and p1-negative the remaining CLL cells. The results, normalized to the GAPDH housekeeping gene and determined by ΔΔCt method, are represented as log10 fold expression ± SD of triplicate assessments. Statistical significance was evaluated using one-way analysis of variance (ANOVA), followed by Bonferroni’s test for multiple comparisons. Bars show mean values ± 95% confidence intervals based on three biological replicates. *P ≤ 0.01; ***P ≤ 0.001; ****P ≤ 0.0001.

Then, we analyzed by qRT-PCR the CD5 expression levels in p1-positive and p1-negative cells, compared to the bulk. As shown in **Figure 3**, a higher expression of CD5 was observed in p1-positive cells (representing the VH 1-69 clone) as compared to p1-negative cells (the remaining CLL clones with IgBCR rearrangements differing from VH 1-69), both in CLL1 (**Figure 3A**) and CLL5 patient (**Figure 3B**), indicating that CD5 expression levels were related to the expression of VH1-69 rearrangement of IgBCR.

## Discussion

CLL clinical course could be characterized by the presence of different tumor B cell clones that could appear or disappear over time, recognized only by the different variable regions of the expressed IgBCRs. These clonal populations may influence the prognosis of the disease, establishing a balanced condition in which the patient remains stable for many years without therapy requirement, or one of them could escape from the apoptosis and proliferation checkpoints, resulting in tumor progression, the need for therapy, and in some cases fatal outcome.

In this scenario, it is interesting the investigation of molecular mechanisms which allow that particular B cell tumor clones to be more aggressive compared to the other tumor populations coexisting in the same patient.

Several studies were conducted in the field of predicting prognosis factors, including the presence or absence of zeta-chain-associated protein kinase (ZAP)-70 or CD38 (21), genomic alterations (10), TP53 status (22), and mutational status of the IgBCR (18).

In particular, the mutational status was one of the first prognostic factors evaluated in CLL patients, observing that patients with unmutated immunoglobulins showed a poor prognosis with drug resistance and rebound (18).

The keystone could be peculiar IgBCR rearrangements that result in self-stimulation through binding to intrinsic motifs of the IgBCR (14, 15) or, on the other side, the presence of a persistent stimulation guided and triggered by endogenous and exogenous epitopes (23–25).

Further, the sequence analysis of IgBCRs expressed by CLL cells, compared to normal B cells, revealed the expression of quasi-identical Ig receptors circa in 30% of diagnosed patients, defined stereotyped IgBCRs (26). These observations fit with the hypothesis of a common exogenous or endogenous antigen stimulating the tumor IgBCRs (14, 15, 23–25).

Reported data describe the capability of the IgBCR to induce the transcription of CD5 by B cells in a murine model (27), which, in turn, transduces pro-survival signaling, such as the IL-10 production (28). Furthermore, it was previously demonstrated that circulating CLL cells, that have been activated recently in proliferating centers, express high levels of surface CD5, which is progressively downregulated as the cells enter into an anergy state, suggesting that the CD5 expression levels could be correlated with the aggressive behavior of the CLL cells (29). Thus, CD5 seems to bind to the Ig heavy chain framework sequence of CLL cells, with a preference for the VH1-69 rearrangement (5), which could promote the selective expansion of B cell clones harboring specific VH genes in CLL.

Our study could represent the proof of concept of the potential use of specific peptide ligands of IgBCRs as probes for sorting and analyzing single tumor subpopulations in CLL patients. Thanks to this new methodology, and according to all well-reported data mentioned above, we observed that the CD5 expression level was increased with the expansion of a specific CLL subpopulation, in our case the unmutated VH1-69 clone, and this could be part of the mechanism of clonal expansion and persistence during the observation, representing additional information for CLL prognosis. More specifically, we demonstrated that single CLL clones could express variable CD5 expression levels, according to their tumorigenic behavior and IgBCR rearrangement.

In perspective, this research line could be extended to all tumor populations of CLL, allowing a wide gene expression analysis, for associating a peculiar IgBCR rearrangement to a specific panel of up or down- regulated genes. We are confident that this approach could get further insights into mechanisms of tumor progression and patient-specific molecular therapy.

## Data Availability Statement

The original contributions presented in the study are included in the article/supplementary materials. Further inquiries can be directed to the corresponding authors.

## Ethics Statement

Experiments involving human subjects were approved by the Italian Regional “Calabria” Ethics Committee (Protocol N. 75, 23/03/17), in accordance with the ethical and safety rules and guidelines provided by the relevant Italian laws (art. 4–5 of D. lgs 116/92, DD.MM. of 29/09/1995 and 26/04/2000), and in accordance with the ethical guidelines of the European Community Council (directive n. 86/609/ECC). Blood samples from healthy donors or CLL patients were obtained upon written and oral informed consent from the participants to the study. The patients/participants provided their written informed consent to participate in this study.

## Author Contributions

DM and SM designed and conducted the research, analyzed the data, and wrote the manuscript. EI assisted with the experiment and data analysis. VD designed qRT-PCR primers and protocols and analyzed data. AD’A, FC, and MG provided CLL samples and clinical analysis. MS performed cell sorting. EV, NN, AA, EDS, and GF focused on data analysis. IQ and SM supervised the research plan and data analysis, and reviewed the manuscript. All authors contributed to the article and approved the submitted version.

## Funding

This work was supported by the following grants: POR FES/FESR 2014-20-ATS ALCMEONE cup J18C17000610006 to IQ; MIUR-PRIN 2017MHJJ55_002 to IQ; GILEAD Fellowship 2018 to EI. SM was supported by funds from the EU project PON-AIM1897004-1; DM was supported by funds from the EU project FSE-FESR PON-RI2014-2020.

## Conflict of Interest

The authors declare that the research was conducted in the absence of any commercial or financial relationships that could be construed as a potential conflict of interest.
